# Prevalence and antimicrobial susceptibility pattern of methicillin resistant *Staphylococcus aureus* isolated from clinical samples at Yekatit 12 Hospital Medical College, Addis Ababa, Ethiopia

**DOI:** 10.1186/s12879-016-1742-5

**Published:** 2016-08-09

**Authors:** Tebelay Dilnessa, Adane Bitew

**Affiliations:** 1 Department of Health Officer, College of Health Sciences, Assosa University, P. O. Box 18, Assosa, Ethiopia; 2 Department of Medical Laboratory Sciences, College of Health Sciences, Addis Ababa University, P. O. Box 1176, Addis Ababa, Ethiopia

**Keywords:** Prevalence, MRSA, MSSA, Beta-lactamase, Penicillin binding protein

## Abstract

**Background:**

*Staphylococcus aureus* particularly MRSA strains are one of the major causes of community and hospital acquired bacterial infections. They are also becoming increasingly multi-drug resistant and have recently developed resistance to vancomycin, which has been used successfully to treat MRSA for many years. In-vitro determination of drug resistance patterns of *S. aureus* is critical for the selection of effective drugs for the treatment of staphylococci infections.

The main aim of this study was to determine the prevalence of methicillin resistant *S. aureus* strains from different clinical specimens from patients referred for routine culture and sensitivity testing.

**Method:**

A cross sectional study was conducted among 1360 participants at Yekatit 12 Hospital Medical College in Ethiopia from September 2013 to April 2014. Clinical samples from various anatomical sites of study participants were cultured on blood agar and mannitol salt agar and identified to be *S. aureus* by using catalase, coagulase and DNAse tests. *S. aureus* isolates then were screened for MRSA using 30 μg cefoxitin disc and other 11 antimicrobial drugs by disc diffusion procedure, and agar dilution and E tests for vancomycin. All *S. aureus* isolates examined for beta-lactamase production by employing nitrocefin. Data were analyzed using SPSS version 20 software and logistic regressions were applied to assess any association between dependent and independent variables.

**Results:**

Of 1360 clinical specimens analyzed *S. aureus* was recovered from (194, 14.3 %). Rate of isolation of *S. aureus* with regard to clinical specimens was the highest in pus (118, 55.4 %).No *S. aureus* was isolated from CSF and urethral discharge. Out of 194 *S. aureus* isolates, (34, 17.5 %) were found out to be MRSA and the remaining (160, 82.5 %) were MSSA. Ninety eight (50.5 %) *S. aureus* were multi drug resistant and the highest isolates were resistant to penicillin (187, 96.4 %) and least resistant for clindamycin (23, 11.9 %) and vancomycin (10, 5.1 %). MRSA strains were 100 % resistant to penicillin G, erythromycin, trimethoprim-sulfamethoxazole and least resistant to vancomycin (10, 29.4 %). Out of 194 *S. aureus* isolates (153, 79.0 %) were beta-lactamase producers.

**Conclusion:**

In this study *S. aureus* isolates exhibited very high degree of resistance to different antibiotics. The isolates were also multidrug resistant to several combinations of the tested antibiotics. The emergence of vancomycin resistant *S. aureus* highlights the value of prudent prescribing of antibiotics and avoiding their irrational use.

## Background

Staphylococcal infections still remain an important cause of mortality and morbidity worldwide despite the development of antimicrobial agents. Among the staphylococcus species, *Staphylococcus aureus* is the most virulent species of the genus causing both nosocomial and community acquired infections worldwide [1]. The organism has been found to be the most common bacterial agent recovered from blood stream infections, skin and soft tissue infections, pneumonia and hospital acquired post operative wound infections [2]. Changes in the drug susceptibility profile of *S. aureus* have been reported worldwide; thereby making treating infections caused by *S. aureus* more difficult [3–5].

Dramatic changes in the susceptibility of *S. aureus* to beta-lactam antibiotics particularly to penicillin and cephalosporin in both hospital and community settings have been reported worldwide [6]. Several mechanisms for the development of MRSA have been reported. Among these production of a unique penicillin-binding protein (PBP) that has a low affinity for β-lactam antibiotics and whose effects are determined by several structural genes (e.g., mecR1 and mecI) [7, 8], production of the usual PBPs, but with modified affinities for β-lactam drugs, and production of penicillinase enzyme are most important ones [9].

MRSA spreads more readily than other strains once introduced into hospitals, and are often difficult to eradicate once established. In some countries MRSA make up to 75 % of all *S. aureus* isolates in hospitals [10]. Transmission of MRSA occurs primarily from colonized or infected patients or staff to other patients or staff, or vice versa [11]. Prevalence, however, varies markedly in hospitals in the same country and from one country to another. Furthermore, in Ethiopia, little information exists regarding prevalence and drug susceptibility pattern of methicillin resistant *S. aureus* isolated from various clinical samples. Therefore, studies on the prevalence and drug susceptibility patterns of *S. aureus* are of the highest priorities.

## Methods

### Study setting and context

A cross sectional study was conducted among 1360 study participants at Yekatit 12 Hospital Medical College, Addis Ababa, Ethiopia from September 2013 to April 2014. The hospital is a tertiary level referral and teaching hospital which provides health care services to patients in and around Addis Ababa, the capital city of the country.

### Specimen collection and processing

Clinical samples were collected from participants by employing standard microbiological procedures. Nasal swab, pus from wound, ear discharge, blood, throat swab, eye swab, vaginal discharge, urethral discharge, urine, stool, sputum, CSF and body fluids were clinical specimens collected. All specimens were transported to microbiology laboratory of the hospital with minimum delay for culture and sensitivity tests.

Clinical specimens were inoculated onto blood agar base (Oxoid, Basingstoke, Hampshire, England) to which 5 % sheep blood was added and mannitol salt agar (Oxoid, Basingstoke, Hampshire, England) by using streaking method. Inoculated plates were incubated at 35–37 °C for 18 to 24 h aerobically. Bacterial colonies showing typical characteristics of *S. aureus* (i.e., beta hemolytic on blood agar and colonies with golden yellow pigmentation on mannitol salt agar) were subjected to subculture on to basic media, gram stain and biochemical tests catalase and coagulase. Catalase positive and gram positive bacteria appearing in grape like cluster was spot inoculated to DNase agar (Oxoid, Basingstoke, Hampshire, England). Inoculated DNAse agar plates were incubated at 37 °C over night and flooded with 1 N HCl (Merk, Darmstadt, Germany). Isolates that hydrolyzed DNA in DNAse agar were considered *S. aureus*.

### Antimicrobial susceptibility testing

Antimicrobial susceptibility test was carried out by Kirby Bauer disc diffusion method as per Clinical Laboratory Standards Institute (CLSI, 2013) guidelines on Muller Hinton agar (Oxoid, Basingstoke, England) for 11 antimicrobials [12]. The growth suspension was prepared in 0.5 ml of the same broth medium and the turbidity was adjusted to match that of 0.5 McFarland standards to obtain approximately the organism number of 1 × 10^6^ colony forming units (CFU) per ml. A sterile swab was dipped into the suspension and the excess of inoculum was removed by pressing it against the sides of the tube. Then the swab was applied to the center of Muller Hinton agar plat and evenly spread on the medium. Antibiotic discs were placed after 15 min of inoculation to Muller Hinton agar seeded with each isolate and were incubated for 24 h at 35–37 °C. The diameter of the zone of inhibition around the disc was measured using sliding metal caliper.

Vancomycin susceptibility test was done by agar dilution and E test (Epsilometer) techniques. Agar dilution test involves the incorporation of varying concentrations of vancomycin into an agar medium, using serial twofold dilutions, followed by the application of a defined *S. aureus* inoculum to the agar surface of the plate. These results are interpreted based on determination of MIC of vancomycin in μg/ml (usually 2 μg/ml to16μg/ml concentration) for *S. aureus*. E test is manufactured by biomerieux, consists of a predefined, continuous and exponential gradient of antibiotic concentrations immobilized along a rectangular plastic test strip. After 48 h incubation a drop-shaped inhibition zone intersects the graded test strip at the inhibitory concentration of the antibiotic. Vancomycin resistant strains were also checked in reference laboratory at EPHI by Broth Microdilution (BMD) method before declaring as resistant. In this broth microdilution, susceptibility panel in 96-well microtiter plates were containing various concentration of antimicrobial agents. Then, standardized numbers of bacteria was inoculated into the wells of 96-well microtiter and incubate overnight at 35 °C. The MIC value was observed as the lowest concentration where no viability was observed in the wells of 96-microwell plates after incubation [13].

### Beta-lactamase production test

All *S. aureus* strains were screened for beta-lactamase production by employing nitocefin, the procedures of Efuntoye et al. [14]. Culture of each isolates was streaked onto sticks impregnated with nitocefin a chromogenic cephalosporin (Unipath Limited, Hampshire, England) that produces a rapid color change from yellow to pink/red when the beta-lactam ring is hydrolyzed by beta-lactamase.

### Ethical approval

The study was conducted after it was ethically reviewed and approved by the Department of Research and Ethical Review Committee of Department of Medical Laboratory Sciences, College of Health Sciences, Addis Ababa University. Ethical clearance was also obtained from Addis Ababa Health Bureau. Then permission was obtained from Yekatit 12 Hospital Medical College. Informed written consent was obtained from participants before data collection. All the information obtained from the study subjects were coded to maintain confidentially. When the participants were found to be positive for *S. aureus,* they were informed by the hospital clinician and received proper treatment.

### Definition of terms

MRSA is defined as zone of inhibition less than or equal to 21 mm on MHA with 30 μg cefoxitin disc seeded with growth suspension of *S. aureus* isolates adjusted to 0.5 McFarland standards [12].

MDR is defined as non-susceptibility to at least one agent in three or more antimicrobial categories [13]. MIC is the lowest concentration of the antimicrobial agent that inhibits visible growth of the organism after a 24-hour incubation period.

### Statistical analysis and quality assurance

The reliability of the study findings were guaranteed by implementing quality control measures throughout the whole processes of laboratory work. *S. aureus* ATCC 25923 is a methicillin susceptible strain was used to check the conditions were favorable for detection of resistance. This strain was obtained from Ethiopian Public Health Institute (EPHI). Data were coded, entered and analyzed using SPSS software version 20 (SPSS INC, Chicago, IL, USA). Binary logistic regression was used to determine the association between *S. aureus* and clinical specimens. Multivariate logistic regressions were used to control confounding factors. *P*-values less than 0.05 were taken as statistically significant.

## Results

### Socio-demographic characteristics of participants

A total of 1360 study participants were enrolled in the present study of which (654, 48.1 %) were males and (706, 51.9 %) females with a sex ratio of 0.93:1. The ages of study subjects ranged from 1 month to 89 years with a mean age of 23.4 ± 0.5 years and median age of 21 ± 0.5 years. Most of study participants were in the age group of 1–14 years (461, 33.9 %).

### Prevalence of *S. aureus*

Of a total of 1360 clinical samples *S. aureus* was isolated from (194, 14.3 %). Males had a higher isolation rate of *S. aureus* than females (106, 16.2 %) versus (88, 12.5 %). Rate of isolation of *S. aureus* was the highest in 15–24 years (46, 21.0 %). The isolation rate of *S. aureus* was not significantly associated with sex [AOR, 95 % CI: 1.05(0.72, 1.53), *p* = 0.771] and age groups (*p* >0.05) (Table 1).

**Table 1:** Association of *S. aureus* in study participants with regard to gender and age group at Yekatit 12 Hospital Medical College from September 2013 to April 2014, Addis Ababa, Ethiopia

Variable		Presence of *S. aureus*		COR(95 % CI)	*P*-value	AOR(95 % CI)	*P*-value
		Yes (N/%)	No (N/%)				
Sex	Male	106(16.2)	548(83.8)	0.73(0.54, 0.99)	0.049	1.05(0.72,1.53)	0.771
	Female	88(12.5)	618(87.5)	1	-	1	-
Age group	<1	9(8.6)	96(91.4)	1.47(0.55, 3.92)	0.434	-	-
	1–14	48(10.4)	413(89.6)	1.19(0.55, 2.54)	0.651	-	-
	15–24	46(21.0)	164(79.0)	0.49(0.23, 1.06)	0.072	-	-
	25–34	38(17.0)	184(83.0)	0.67(0.31, 1.46)	0.315	-	-
	35–44	26(16.8)	129(83.2)	0.68(0.30, 1.55)	0.366	-	-
	45–64	18(13.6)	115(86.4)	0.88(0.37, 2.08)	0.779	-	-
	> = 65	9(12.2)	65(87.8)	1	-	-	-

*COR* crude odds ratio, *AOR* adjusted odds ratio, *CI* Confidence interval

### Isolation rate of *S. aureus* from clinical samples

In current study the major sources of *S. aureus* were pus/abscess, ear discharge, blood, nasal swab and throat swab which together, accounted for (175, 90.2 %) of all isolates. The rest of the isolates were from urine, vaginal discharge, eye swab, body fluid, stool and sputum, accounting less than 10.0 % of the total. No *S. aureus* was isolated from CSF (0/97) and urethral discharge (0/19). The recovery rate of *S. aureus* was significantly associated with pus [AOR, 95 % CI: 67.07(9.1, 493.8), *p* = 0.001], nasal swab [AOR, 95 % CI: 27(3.19, 228.05), *p* = 0.002], throat swab [AOR, 95 % CI: 11.1(1.3, 94.37), *p* = 0.027] and ear discharge [AOR, 95 % CI: 21.2(2.8, 162.3), *p* = 0.003] (Table 2).

**Table 2:** Isolation rate of *S. aureus* from clinical specimens at Yekatit 12 Hospital Medical College from September 2013 to April 2014, Addis Ababa, Ethiopia

Specimen type	Presence of *S. aureus*		Total	AOR	95 % CI	*P*-value
	Yes (N/%)	No (N/ %)	N^a^			
Pus/abscess	118(55.4)	95(44.6)	213	67.074	(9.11, 493.8)	0.001^**^
Nasal swab	9(33.3)	18(66.7)	27	27.000	(3.19, 228.05)	0.002^**^
Throat swab	7(17.0)	34(83.0)	41	11.118	(1.31, 94.37)	0.027^*^
Vaginal discharge	3(2.5)	118(97.5)	121	1.373	(0.14, 13.50)	0.786
Urine	6(2.8)	211(97.2)	217	1.536	(0.18, 13.02)	0.694
Eye swab	2(12.5)	14(87.5)	16	7.714	(0.65, 91.32)	0.105
Blood	17(7.6)	206(92.4)	223	4.456	(0.58, 34.23)	0.151
Body fluids	4(5.3)	71(94.7)	75	3.042	(0.33, 28.0)	0.326
Stool	3(1.8)	168(98.2)	171	0.964	(0.09, 9.46)	0.975
Ear discharge	24(28.2)	61(71.8)	85	21.246	(2.78, 162.3)	0.003^**^
Sputum	1(0.8)	54 (98.2)	55	1	-	-

*AOR* adjusted odds ratio

^a^ Total sample

^**^ Significant at *P* value < 0.01, ^*^ Significant at *P* value < 0.05

### Prevalence of MRSA

**Fig. 1:**
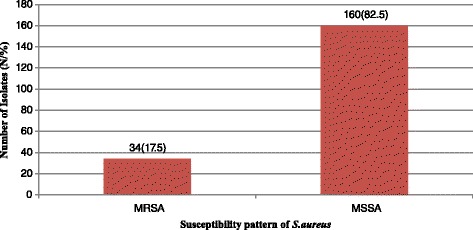
Methicillin susceptibility pattern of *S. aureus* from participants at Yekatit 12 Hospital Medical College from September 2013 to April 2014, Addis Ababa, Ethiopia

Out of 194 *S. aurues* recovered, (34, 17.5 %) were found out to be MRSA and the remaining (160, 82.5 %) were MSSA (Fig. 1). Relatively a higher number of MRSA were isolated in males (19, 19.9 %) than in females (15, 17.0 %) and the highest number of MRSA were detected in the age group 35–44 years (8, 30.8 %), followed by age group 45–64 years (5, 27.8 %), above 64 years (2, 22.2 %), 15–24 years (7, 17.4 %), 1–14 years (7, 14.6 %), and 25–34 years (4, 10.5 %). The isolation rate of MRSA in relation to gender was not significantly associated [COR, 95 % CI: 0.94(0.44, 1.98), *p* = 0.87] as well as any of age groups (*p* >0.05) (Table 3).

**Table 3:** Association of methicillin resistant pattern of *S. aureus* in study participants with gender and age group at Yekatit 12 Hospital Medical College from September 2013 to April 2014, Addis Ababa, Ethiopia

Variable		MRSA	MSSA	*S. aureus*	COR	95 % CI	*P*-value
		N (%)	N (%)	N			
Sex	Male	19(19.9)	87(82.1)	106	0.94	(0.44, 1.98)	0.873
	Female	15(17.0)	73(83.0)	88	1	-	-
Age group	<1	0(0)	9(100)	9	0.00	-	0.999
	1–14	7(14.6)	41(85.4)	48	1.67	(0.29, 9.76)	0.567
	15–24	8(17.4)	38(82.6)	46	1.36	(0.24, 7.78)	0.732
	25–34	4(10.5)	34(89.5)	38	2.43	(0.37, 15.95)	0.356
	35–44	8(30.8)	18(69.2)	26	0.64	(0.11, 3.80)	0.626
	45–64	5(27.8)	13(72.2)	18	0.74	(0.11, 4.86)	0.757
	> = 65	2(22.2)	7(77.8)	9	1	-	-

*COR* crude odds ratio

The major source of MRSA was pus (24/118), nasal swab (3/9), ear discharge (3/24), throat swab (2/7) and blood (2/17). No MRSA was observed in urine, stool, body fluid, eye swab, sputum and vaginal discharge. Blood (15, 88.2 %) had higher percentage of MSSA followed by ear discharge (21, 87.5 %) and pus (94, 79.7 %). There was no statistical association between isolation rates of MSRA with any of clinical samples (*p* > 0.05) (Table 4).

**Table 4:** Association of methicillin resistant *S. aureus* to different clinical specimens at Yekatit 12 Hospital Medical College from September 2013 to April 2014, Addis Ababa, Ethiopia

Type of specimen	MRSA	MSSA	Total	COR	95 % CI	*P*-value
	N (%)	N (%)	N^a^			
Pus/abscess	24(20.3)	94(79.7)	118	0.511	(0.11, 2.19)	0.366
Nasal swab	3(33.3)	6(66.7)	9	0.638	(0.11, 3.49)	0.605
Throat swab	2(28.6)	5(71.4)	7	0.000	-	0.999
Vaginal discharge	0(0)	3(100)	3	0.000	-	0.999
Urine	0(0)	6(100)	6	1.915	(0.41, 8.95)	0.409
Eye swab	0(0)	2(100)	2	0.000	-	0.999
Blood	2(11.8)	15(88.2)	17	0.000	-	0.999
Body fluids	0(0)	4(100)	4	0.000	-	0.999
Stool	0(0)	3(100)	3	0.000	-	1.000
Ear discharge	3(12.5)	21(87.5)	24	1.787	(0.49, 6.49)	0.378
Sputum	0(0)	1(100)	1	1	-	-

*COR* crude odds ratio

^a^ total *S. aureus* isolates from each type of sample

### Antimicrobial susceptibility pattern of *S. aureus*

*S. aurues* isolated in this study were highly resistant to penicillin (187, 96.4 %), trimethoprim-sulphamethoxazole (103, 53.1 %), erythromycin (103, 53.1 %) and ciprofloxacin (61, 31.4 %). On the contrary, lower resistant was manifested by amoxicillin-clavulanate (36, 18.5 %), gentamicin (26, 13.4 %), clindamycin (23, 11.9 %) and vancomycin (10, 5.1 %) (Table 5).

**Table 5:** Antimicrobial susceptibility pattern of *S. aureus* strains to different antimicrobial agents at Yekatit 12 Hospital Medical College from September 2013 to April 2014, Addis Ababa, Ethiopia

Antibiotics	Resistant	Susceptible
	N (%)	N (%)
Cefoxitin [30 μg]	34(17.5)	160(82.5)
Amoxicillin-clavulanate [30 μg]	36(18.5)	158(81.5)
Penicillin G [10 U]	187(96.4)	7(3.6)
Vancomycin^a^	10(5.1)	184(94.9)
SXT [1.25/23.75 μg]	103(53.1)	91(46.9)
Chloramphenicol [30 μg]	36(18.6)	158(81.4)
Gentamycin [10 μg]	26(13.4)	168(86.6)
Cefuroxime [30 μg]	40(20.6)	154(79.4)
Clindamycin [30 μg]	23(11.9)	171(88.1)
Ciprofloxacin [5 μg]	61(31.4)	133(68.6)
Cephalothin [10 μg]	37(19.1)	157(80.9)
Erythromycin [15 μg]	103(53.1)	91(46.9)

*SXT* Trimethoprim-Sulfamethoxazole

^a^ concentration from 2 μg/ml to16μg/ml

All isolates were resistant to at least one antimicrobial agent. MRSA isolates were 100 % resistant for penicillin, erythromycin, trimethoprim-sulfamethoxazole, amoxicillin-clavulanate, cefuroxime, cephalothin and least resistant for vancomycin (10, 29.4 %). On the other hand, (153, 95.6 %) of MSSA were resistant to penicillin. Being resistant/susceptible for methicillin had a statistically significant chance of being resistant/susceptible for amoxicillin-clavulanate, cefuroxime and clindamycin (Table 6).

**Table 6:** Association of methicillin resistant and sensitive *S. aureus* to different antimicrobial classes at Yekatit 12 Hospital Medical College from September 2013 to April 2014, Addis Ababa, Ethiopia

Antibiotics	MRSA (*n* = 34)	MSSA (*n* = 160)	AOR	95 % CI	*P*-value
	N (%)	N (%)			
AMC [30 μg]	34(100)	2(1.3)	9.809	(1.56, 61.69)	0.015^*^
Penicillin G [10 U]	34(100)	153(95.6)	0.000	-	0.99
Vancomycin^a^	10(29.4)	0(0)	0.000	-	0.99
SXT [1.25/23.75 μg]	34(100)	69(43.1)	16.24	(0.41, 63.28)	0.135
Chloramphenicol [30 μg]	16(47)	20(12.5)	1.130	(0.09, 13.63)	0.923
Gentamycin [10 μg]	13(38.2)	13(8.1)	0.896	(0.07, 10.98)	0.931
Cefuroxime [30 μg]	34(100)	6(3.8)	216.17	(10.8, 432.2)	0.001^**^
Clindamycin [30 μg]	18(53)	5(3.1)	13.22	(1.99, 87.62)	0.007^**^
Ciprofloxacin [5 μg]	28(82.5)	33(20.6)	0.615	(0.10, 3.73)	0.598
Erythromycin [15 μg]	34(100)	69(43.1)	9.044	(0.49, 16.05)	0.136
Cephalothin [10 μg]	34(100)	3(1.8)	1	-	-

*AMC* Amoxilin- clavulanic acid, *SXT* Trimethoprim-Sulfamethoxazole

^a^ concentration from 2 μg/ml to16μg/ml

^**^ Significant at *P* value < 0.01, ^*^ Significant at *P* value < 0.05

### Multi-drug resistance (MDR) and Beta-lactamase production pattern of *S. aureus*

Ninety-eight (50.5 %) of the isolates were multi-drug resistance. According to Magiorakos et al. [15] MDR is defined as non-susceptibility to at least one agent in three or more antimicrobial categories. A higher number of multi-drug resistances were observed in triple drugs among penicillin G, erythromycin and trimethoprim-sulfamethoxazole (18, 18.4 %), and quadruple drugs among penicillin G, erythromycin, trimethoprim-sulphamethoxazole and ciprofloxacin (13, 13.26 %). Thirty (30.64 %) of multi-drug resistance was observed by triple drugs. Penicillin resistant was seen in all multi-drug resistant strains of *S. aureus* (Table 7). Of 194 *S. aureus* isolates, (153, 79.0 %) were beta-lactamase producers. Furthermore, of 34 MRSA isolates (30, 88.2 %) and out of 160 MSSA strains (123, 76.8 %) were produced beta-lactamase.

**Table 7:** Multi*-*drug resistance nature of *S. aureus* isolates at Yekatit 12 Hospital Medical College from September 2013 to April 2014, Addis Ababa, Ethiopia

Antibiotics	Resistant strains	
	N	%
P, E, SXT	18	18.40
P, G, E	5	5.10
P, C, SXT	4	4.08
P, C, CIP	3	3.06
P, CIP, E, SXT	13	13.26
P, G, C, E	3	3.06
P, G, C, SXT	2	2.04
P, CIP, E, SXT	1	1.02
P, AMC, E, SXT	1	1.02
P, FOX, G, SXT	1	1.02
P, FOX, E, SXT	2	2.04
P, C, CIP, E, SXT	2	2.04
P, G, CIP, AMC, SXT	1	1.02
P, G, CIP, E, SXT	1	1.02
P, G, C, E, SXT	1	1.02
P, CIP, E, SXT, DA	2	2.04
P, FOX, C, E, SXT	1	1.02
P, G, C, CIP, CXM, SXT	1	1.02
P, G, C, CIP, E, SXT	3	3.06
P, G, C, AMC, E, SXT	1	1.02
P, FOX, CIP, E, SXT, DA	3	3.06
P, FOX, C, E, CXM, SXT	2	2.04
P, G, C, CIP, E, SXT, DA	2	2.04
P, FOX, G, C, CIP, E, SXT	2	2.04
P, FOX, C, CIP, E, CXM, SXT	5	5.10
P, FOX, C, E, CXM, SXT, DA	1	1.02
P, FOX, CIP, AMC, KF, E, CXM	2	2.04
P, FOX, CIP, AMC, KF, SXT, DA	2	2.04
P, FOX, G, C, CIP, E, CXM, SXT	2	2.04
P, FOX, VAN, CIP,AMC, E, SXT, DA	5	5.10
P, FOX, VAN, CIP, AMC, E, CXM, SXT, DA	1	1.02
P, FOX, C, CIP, AMC, E, CXM, SXT, DA	1	1.02
P, FOX, VAN, CIP, AMC, KF, E, SXT, DA	1	1.02
P, FOX, VAN, G, C, CIP, AMC, E, SXT, DA	1	1.02
P, FOX, VAN, G, C, CIP, AMC, E, CXM, SXT, DA	2	2.04
Total	98	100.00

*P* Penicillin G, *CIP* Ciprofloxacin, *AMC* Amoxicillin-clavulanic acid, *C* Chloramphenicol, *FOX* Cefoxitin, *KF* Cephalothin, *E* Erythromycin, *G* Gentamycin, *VAN* Vancomycin, *SXT* Trimethoprim-sulphamethoxazole, *DA* Clindamycin, *CXM* Cefuroxime

## Discussion

The present study showed that males had a higher isolation rate of *S. aureus* than females. Rate of isolation of *S. aureus* was also the highest in 15–24 years of age group. Prevalence of MRSA in the present study, however, did not vary significantly by gender (*p* = 0.87) and age group (*p* > 0.05) and this is in agreement with earlier reports by Geyid et al. [16] indicating that gender and age are not risk factor for the acquisition or colonization of MRSA. The prevalence of MRSA was found to be 17.5 % which is less than that had been reported in Addis Ababa [16, 17] and outside Addis Ababa [18]. Different studies have depicted variations in the prevalence rates of MRSA in different countries. Over 50 % prevalence rate of MRSA was reported in Portugal and Italy; 25 % in England, Greece and France; 2 % in the Netherlands and Switzerland [19]. Prevalence of MRSA ranged from 23.6 % in Australia to over 61 % in Taiwan and Singapore, and more than 70 % in Japan and Hong Kong [20]. Differences in the length of study period, number of study sites, sample size, sample type and the laboratory procedures employed may be factors that could contribute to variations in the prevalence rate of MRSA [21]. The rate of MRSA obtained in this study however, was nearly the same as MRSA prevalence rate recorded in a pan-European data that was obtained from studies conducted among 43 laboratories from 10 European countries [22].

Although no statistical association existed between isolation rates of MSRA and MSSA with any of clinical samples (*p* >0.05), the present study depicted that prevalence of MRSA and MSSA isolated from pus was the highest as compared to other clinical samples. This finding is in agreement with the result obtained in Ethiopia [16, 18] and many similar studies [21, 23, 24] conducted in other parts of the world. A highest isolation rate of *S. aureus* in general and MRSA in particular in pus in our study could partly be due to the fact that most of the wound samples came from surgical wards and burn unit of the hospital. MRSA on surgical wards is becoming increasingly common especially in critically ill patients who have spent prolonged periods on the intensive care units [22, 23].

Even though this study was not designed to identify risk factors for MRSA acquisition, risk factors that have previously been associated with acquisition of MRSA in hospitals such as broad-spectrum antimicrobial therapy, admission to an intensive care unit, older age and proximity to other patients with MRSA [25] could play a major role in our study site. Antimicrobial susceptibility test on all the 194 *S. aureus* isolates against 12 commonly used antibiotics indicated that (187, 96.4 %) were resistant to penicillin and this finding was in agreement with the findings of Abera et al. [18]. The lowest drug resistant was observed for vancomycin (10, 5.1 %). Furthermore, (98, 50.5 %) of the isolates were multi-drug resistant which is consistent with a study in Ethiopia at Bahir Dar [18]. But it was less than the results reported at Addis Ababa [16]. This variation could be explained in terms of periods of study, hygienic practice of the population, difference in socio-cultural and economic activity of the community.

All MRSA isolates encountered in this study were completely resistant (100 %) to antibiotics such as penicillin, erythromycin and trimethoprim-sulfamethoxazole. Similar results were noted for penicillin among MRSA strains in India [26] and Trinidad & Tobago [21]. Unlike most studies in Ethiopia [16, 17] and elsewhere in the world [21, 27] vancomycin resistance was very high. Ten (5.1 %) out of 194 *S. aureus* isolates were resistant to vancomycin and of 34 MRSA, (10, 29.4 %) were vancomycin resistant. A similar result was obtained for vancomycin resistance in previous reports in Trinidad [28], and Argentina, Brazil, Chile, Mexico and Uruguay [29]. MRSA strains which were also resistant to vancomycin in previous studies ranged from none (0 %) in Ethiopia, Karachi and Uganda [16, 27, 30] to 8 % in Iran, Malaysia and Nigeria [31–33] which are lower than the current study. A study in United States and Latin American countries reported that most MRSA strains are resistant to other antibiotics, thereby necessitating the use of glycopeptides antibiotics, such as vancomycin. Treatment failure has been incriminated as a cause of decreased susceptibility of staphylococci to vancomycin [34]. Wise use and continuous surveillance susceptibility testing of MRSA against vancomycin have been reported as a remedy to control reduced susceptibility of staphylococci to vancomycin [35–37].

In the present study all *S. aureus* isolates were tested for beta-lactamase production. It has been shown that out of 194 isolates (153, 79.0 %) were beta-lactames producers. Beta-lactamase producing strains of *S. aureus* in the present study were much higher than that has been reported by previous study conducted in Ethiopia [16] but more or less the same as reported in India [38] and Nigeria [14]. Our study further depicted that out of 34 MRSA strains (30, 88.2 %) produced beta-lactamase and out of 160 MSSA strains (123, 76.8 %) were found out to be beta-lactamase producers.

## Conclusion

The prevalence of *S. aureus* and MRSA varies appreciably based on type of clinical samples. Pus was the main source of *S. aureus* and MRSA in the hospital settings. The prevalence of MRSA stains obtained in this study was low when compared with the prevalence rates obtained in previous studies conducted in Ethiopia. However, the prevalence rate was considerably high when compared to other similar studies conducted elsewhere. MRSA strains were multidrug-resistant and unusually high numbers of isolates were resistant to vancomycin, the drug of choice for treating multidrug resistant MRSA infections. Reducing this burden by good infection control practices such as strict hand washing, identifying MRSA carriers and treating them as well as prudent use of antimicrobial agents is recommended. Further, genotypic studies are also needed to establish and characterize resistant strains of *S. aureus*.

## Abbreviations

AAU, Addis Ababa University; AST, antimicrobial susceptibility testing; CLSI, Clinical and Laboratory Standards Institute; CSF, cerebrospinal fluid; EPHI, Ethiopian Public Health Institute; MDR, multi-drug resistance; MHA, Muller Hinton Agar; MIC, minimum inhibitory concentration; MRSA, methicillin resistant *Staphylococcus aureus*; MSSA, methicillin sensitive *Staphylococcus aureus*; PBP, penicillin binding protein
